# The Development of Attentional Biases for Faces in Infancy: A Developmental Systems Perspective

**DOI:** 10.3389/fpsyg.2018.00222

**Published:** 2018-02-28

**Authors:** Greg D. Reynolds, Kelly C. Roth

**Affiliations:** Developmental Cognitive Neuroscience Laboratory, Department of Psychology, University of Tennessee, Knoxville, TN, United States

**Keywords:** infancy, social development, attentional biases, perceptual processing, event-related potentials

## Abstract

We present an integrative review of research and theory on major factors involved in the early development of attentional biases to faces. Research utilizing behavioral, eye-tracking, and neuroscience measures with infant participants as well as comparative research with animal subjects are reviewed. We begin with coverage of research demonstrating the presence of an attentional bias for faces shortly after birth, such as newborn infants’ visual preference for face-like over non-face stimuli. The role of experience and the process of perceptual narrowing in face processing are examined as infants begin to demonstrate enhanced behavioral and neural responsiveness to mother over stranger, female over male, own- over other-race, and native over non-native faces. Next, we cover research on developmental change in infants’ neural responsiveness to faces in multimodal contexts, such as audiovisual speech. We also explore the potential influence of arousal and attention on early perceptual preferences for faces. Lastly, the potential influence of the development of attention systems in the brain on social-cognitive processing is discussed. In conclusion, we interpret the findings under the framework of Developmental Systems Theory, emphasizing the combined and distributed influence of several factors, both internal (e.g., arousal, neural development) and external (e.g., early social experience) to the developing child, in the emergence of attentional biases that lead to enhanced responsiveness and processing of faces commonly encountered in the native environment.

## Introduction

Developing the ability to process and respond appropriately to social stimuli is critically important for social, cognitive, and emotional development. Attentional biases refer to a readiness to orient toward and maintain attention on a particular class of stimuli over others. These biases can be driven by mechanisms associated with increased probability of rapid orienting to certain types of stimuli and/or mechanisms involved in a lower probability of disengaging attention from certain types of stimuli (e.g., Cohen, 1972; Posner et al., 1987; Pool et al., 2016). Research indicates that in certain contexts even newborn infants possess attentional biases to orient toward and maintain visual fixation on faces and face-like stimuli over non-face stimuli (e.g., Johnson and Morton, 1991b; Morton and Johnson, 1991; Pascalis et al., 1995; Valenza et al., 1996). These biases likely facilitate social responsiveness and perceptual learning in the earliest stages of postnatal development.

Controversy has arisen in the field over potential mechanisms that may account for these early emerging attentional biases. Some have argued in support of domain-general mechanisms that are driven by a match between low-level stimulus characteristics of face-like stimuli and stimulus properties that attract orienting and visual fixation of the developing newborn visual system (e.g., Kleiner and Banks, 1987; Gauthier and Nelson, 2001; Macchi Cassia et al., 2001; Nelson, 2001; Simion et al., 2001; Turati, 2004). In contrast, others have argued in support of domain-specific mechanisms associated with evolved neural systems dedicated to face detection (e.g., Johnson and Morton, 1991b; Farroni et al., 2006; Pascalis and Kelly, 2009; Johnson et al., 2015). Understanding the origins of attentional biases is of the upmost importance as the distribution of selective attention plays of fundamental role in early perceptual learning that may have cascading effects on subsequent cognitive development (e.g., Reid and Striano, 2007; Bahrick and Lickliter, 2014; Amso and Scerif, 2015; Reynolds, 2015).

Developmental systems theorists have proposed that phenotypic outcomes are the product of reciprocal and bidirectional interactions of multiple factors both internal and external to the developing organism (e.g., Gottlieb, 1991, 2007; Thelen, 1992; Thelen and Smith, 1994; Lickliter, 1996, 2000; Oyama et al., 2001; Gottlieb et al., 2006; Lewkowicz, 2011). In this paper, we review research on the development of attentional biases for faces in infancy. Under the framework of developmental systems theory, we propose that multiple factors influence the development of attentional biases, and these biases subsequently guide infant selective attention and perceptual learning in social contexts. We begin by reviewing research on early emerging perceptual preferences for faces and face-like stimuli over non-social stimuli, and the potential role of prior experience on attentional biases for faces. We subsequently review research on the development of face processing in multimodal contexts, followed by a section on physiological and neural mechanisms that are likely associated with attentional biases in infancy. The purpose of this paper is not to present exhaustive reviews of the extant literature in each of these areas of research. Instead, we review relevant findings and developmental theory for the purpose of building a conceptual framework for understanding potential mechanisms involved in the early development of attentional biases for faces. We propose that the attentional biases newborns demonstrate for face-like stimuli, and the relatively rapid developmental changes that occur in face processing during infancy can be explained under the framework of developmental systems theory through application of the following domain-general developmental principles: *constraints by design* (Lickliter, 2000; Lickliter and Harshaw, 2010), *experiential canalization* (Kuo, 1976; Gottlieb, 1991; Lickliter and Harshaw, 2010), and *distributed control* (Lickliter, 2000; Lickliter and Honeycutt, 2003).

## Attentional Biases for Faces in Newborns

Research has shown that even newborn infants prefer faces and face-like stimuli over non-social stimuli (e.g., Johnson et al., 1991a; Pascalis et al., 1995; Macchi Cassia et al., 2001). Several factors specific to faces have been shown to influence newborn attentional biases. For example, when viewing faces, newborns demonstrate visual preferences for: open-eyes compared to closed-eyes (Batki et al., 2000), attractive faces compared to less attractive faces (Slater et al., 1998, 2000a,b), direct compared to averted eye gaze (Farroni et al., 2002), and mother’s face compared to a stranger’s face (Field et al., 1984; Pascalis et al., 1995). Newborn attentional biases generalize to face-like patterns, such as top-heavy inverse triangles (Valenza et al., 1996; Simion et al., 2002), but by 3 months of age, infants only demonstrate preferences for faces, and the preference for top-heavy geometric patterns no longer exists (Chien, 2011). Taken together, these findings indicate an early preference for visual patterns that structurally resemble the human face (the top-heavy or inverse triangle pattern), which then progresses to a more specific preference for human faces that is likely tied to extensive early social experience and exposure to human faces which we discuss in more detail in a later section

The presence of an attentional bias for faces and face-like geometric patterns in the newborn period has led to substantial controversy and debate in the literature on the development of face processing. This debate revolves around differing theoretical views on potential mechanisms that may account for the presence of this attentional bias in newborns who have had highly limited postnatal visual experience with faces. Several models have been proposed that attempt to explain the presence of newborn attentional biases for faces as well as the rapid development of face processing across infancy. The two-process theory of face processing proposed by Johnson and Morton (1991b), Morton and Johnson (1991), and recently modified by Johnson et al. (2015) is one of the most influential theoretical models to date. According to this model, there are two systems involved in face processing. Conspec is the first system, which is a subcortical system involved in orienting to faces of conspecifics. This system accounts for newborn attentional biases for faces and face-like stimuli. The second system, Conlern, represents an acquired system of cortical circuits involved in processing faces (i.e., face recognition, categorization, etc.) that is influenced by experience and learning. An important component of this model is that Conspec is an innate system which serves to bias infant visual orienting toward faces of conspecifics, thus insuring appropriate input for further development and specialization of neural systems associated with Conlern. Neuroanatomical structures associated with Conspec include areas involved in the retino-tectal visual pathway: the superior colliculus, pulvinar, and amygdala complex (Johnson et al., 2015). Neuroanatomical structures involved in Conlern include: the fusiform gyrus, superior temporal sulcus, medial prefrontal cortex, and orbitofrontal cortex. Support for the proposal that newborn attentional biases are driven by a subcortical circuit come from findings indicating that newborns only show preferences for face-like stimuli when the stimuli are presented in the temporal visual field (Simion et al., 1998), which feeds differentially into the subcortical visual pathway (Sylvester et al., 2007).

There are several influential alternative models to the two-process theory of face processing. Slater (1993), Slater et al. (2010) have proposed a model based on Valentine’s (1991) concept of Face Space. Similar to the two-process theory, this model posits that an attentional bias exists at birth for infants to orient toward face-like stimuli, and that experience with faces shapes the face processing system as a prototypical face space is constructed based on dimensions (e.g., age, attractiveness, race, species) that serve to discriminate the types of faces commonly encountered in the native environment. In contrast to Conspec (Johnson and Morton, 1991b; Johnson et al., 2015), Slater et al. (2010) propose that the initial bias to orient toward faces in the newborn period is not innate, and many of the properties of faces that attract newborn attention are not specific to faces. Slater et al. (2010) further propose that newborns demonstrate more advanced face processing consistent with rapid development of face space than the basic orienting functions served by Conspec. Evidence in support of their position comes from the finding that newborns demonstrate visual preferences for attractive faces. This effect is driven by attention to internal facial features and only found when newborns view upright faces (Slater et al., 2000a,b). Inversion effects are related to configural processing and developing expertise in face processing that would not be expected to occur if face processing was exclusively under the control of a subcortical system (i.e., Conspec). Further support for the possibility that cortical structures are involved in early face processing comes from a study conducted by Nakano and Nakatani (2014) using S-cone isolating stimuli which are processed exclusively by the geniculo-cortico visual pathway. Two-month-old infants were found to show a preference for upright compared to inverted S-cone isolating face stimuli indicating relatively advanced cortical processing of face stimuli by the end of the newborn period. Quinn and Slater (2003), Slater et al. (2010) have proposed that newborn attentional biases for faces may be the product of general properties of the nervous system, gestational proprioceptive feedback, and face-specific biases.

The possibility that attentional biases in the newborn period are likely influenced by multiple factors, including general properties of the nervous system and prenatal sensory experience, is in line with developmental systems theory and the concept of *constraints by design* (Lickliter, 2000; Lickliter and Harshaw, 2010; also see, Oyama, 1993). According to this concept, both the buffered nature of the prenatal environment as well as the relatively immature sensory capacities of the developing organism provide constraints that limit early sensory experience. Neonatal sensory limitations also serve as a source of perceptual organization in early development under this framework (Turkewitz and Kenny, 1982; Lewkowicz, 2000). The structure provided by these external and internal factors provides an orderly and consistent context for development and can serve as a source of stability in species-typical perceptual development (Lickliter, 1996, 2000). The concept of constraints by design is similar to the concept of global determinism, which is the notion that internal and external boundaries of developmental systems provide stability in outcomes that emerge in the process of development (Thelen, 1992).

In line with the concept of constraints by design, Simion et al. (2001) proposed that domain-general perceptual constraints on visual processing account for newborn attentional biases for faces and face-like patterns as opposed to a domain-specific innate facial representation. Studies providing support for this position have shown top heavy configural patterns can elicit a stronger visual preference in newborns than a schematic face (Easterbrook et al., 1999; Turati et al., 2002). Similarly, newborns’ visual preferences have been found to be influenced by: the amplitude spectrum of the stimulus (Mondloch et al., 1999), contrast-polarity (Farroni et al., 2005), up-down asymmetry (Simion et al., 2002), and congruency between internal and boundary elements (Macchi Cassia et al., 2008). Taken together, these findings support the possibility that newborn attentional biases for faces are based on stimulus characteristics that are not unique to faces but do fall within an optimal range for visual processing given domain-general perceptual constraints of the immature visual system.

Wilkinson et al. (2014) proposed the binocular correlation model (BCM), which is arguably the most well-formulated model of newborn face preferences proposed to date. The model is based on an extension of the linear systems model (Banks and Salapatek, 1981; Banks and Ginsburg, 1985). The linear systems model has been used to model the filtering properties of the immature visual system based on factors such as the resolution of rods and cones, and the average contrast sensitivity function in order to quantify the visibility of stimuli for the newborn infant. Infant visual preferences are proposed to be proportionately related to the visibility of the stimuli being tested. The BCM extends the linear systems model from a monocular to a binocular visual system. The model also includes the addition of a factor of facial embodiment, such that the spacing of the eyes (i.e., inter-pupillary distance) is mapped to the lateral geniculate nucleus, thus providing structural information regarding faces to the visual system. The model predicts that infants will prefer to look at stimuli that result in stronger correlations between corresponding points in the visual arrays of both eyes, and that faces and face-like patterns will result in high levels of binocular correlation because of the match between inter-pupillary distance on the infant’s face and salient areas of face stimuli that are likely fixation points (e.g., the eyes on the fixated face). One of the greatest strengths of this model is that it can be tested, and the authors (Wilkinson et al., 2014) did so by utilizing computational modeling in tandem with a humanoid robot. Through a series of simulations, the robot’s looking to face-like patterns used in previous research on newborn face preferences was tested. Results from the modeling demonstrated consistency with newborn attentional biases found in previous studies and provided support for the BCM. A major advantage of the BCM is that, unlike the two-process theory of infant face processing, it does not rely on the existence of an innate representation of face information. Another strength of this model is the extension of linear systems model to binocular vision characteristic of the human visual system. A limitation of the current model is that it fails to replicate inversion effects often seen in newborns. However, Wilkinson et al. (2014) acknowledged that newborn facial processing is likely affected by more than just binocular correlation, and the addition of a bias for upper visual field salience (e.g., Easterbrook et al., 1999; Turati et al., 2002) would likely increase the accuracy of the model.

In addition to structural characteristics of the face and visual capacities of the newborn, it is possible that prenatal experience biases newborn infants to attend to certain types of social stimuli. In support of this possibility, findings from both comparative and human research have shown that recently hatched Bobwhite Quail chicks (e.g., Lickliter et al., 2002) and human newborns (DeCasper and Fifer, 1980; DeCasper and Spence, 1986; Fifer and Moon, 1989) demonstrate familiarity preferences for specific auditory stimuli they were exposed to during the late stages of prenatal development. Furthermore, human newborns prefer their mother’s native language (i.e., maternal language) over non-maternal language (Spence and DeCasper, 1987; Moon et al., 1993), and newborns show evidence of detecting changes in affect in speech conveyed in their maternal language but do not show such evidence of affect discrimination in non-maternal language (Mastropieri and Turkewitz, 1999). These findings demonstrate effects of prenatal auditory experience on postnatal perceptual responsiveness resulting in increased sensitivity to stimuli encountered in the prenatal period. Lickliter (1994), Markham et al. (2008) have also shown that prenatal sensory stimulation in one sensory modality (e.g., visual) can influence subsequent postnatal perceptual responsiveness in another sensory modality (e.g., auditory).

It is possible that prenatal auditory stimulation could have similar effects on postnatal visual responsiveness and attentional biases in human development. For example, frequent exposure to the mother’s voice during prenatal development followed by exposure to the mother’s voice paired with her face shortly after birth could contribute to rapid development of visual preferences for human faces. These biases may then serve to facilitate selective attention to faces and voices in the newborn period. Although this possibility is speculative and remains untested, a series of studies conducted by Sai (2005) demonstrated the influence of maternal speech on newborn attentional biases for faces. Newborn infants who were exposed to their mother’s speech between birth and testing preferred their mother’s face over a stranger’s face. In contrast, newborns who had no postnatal exposure to their mother’s speech prior to testing showed no visual preference for their mother’s face over a stranger’s face. Although carrying out a systematic empirical investigation on the effects of prenatal sensory experience on postnatal visual responsiveness seems impractical at best, computational modeling could potentially be utilized to test the feasibility of the impact of prenatal sensory experience on visual responsiveness in the postnatal period. For example, Bednar and Miikkulainen (2006) utilized computational modeling to demonstrate that newborn face biases could be influenced by internally generated input patterns provided by ponto-geniculo-occipital waves that occur during REM sleep in the prenatal period. The authors proposed that the combined influence of prenatal learning and internal patterns could contribute to the development of neural circuitry involved in face processing.

In a recent exploratory study, Reid et al. (2017) utilized 4-D ultrasound technology to image fetal movements while projecting an upright or inverted triangle pattern composed of three lights through the mother’s abdomen. The ultrasound was used to identify the location of the fetus, and the triangular pattern of lights was either projected inverted (i.e., top-heavy) or upright (bottom-heavy) relative to the fetal position. Fetal movements following the presentation of the light pattern were measured as an index of orienting, and more fetal movements were found during presentations of the inverted (face-like) triangle pattern of lights. The authors interpreted this as indicating postnatal experience is not necessary for explaining newborn preference for face-like patterns. This study represents an important step toward developing techniques for prenatal testing with human fetuses. However, given the highly exploratory nature of this study, validation and replication are needed before making strong conclusions based on these results.

The utilization of computational modeling (Bednar and Miikkulainen, 2006; Wilkinson et al., 2014) has provided some insight into potential mechanisms involved in newborn attentional biases for faces. In our opinion, the BCM (Wilkinson et al., 2014) provides the strongest model proposed to date for explaining attentional biases for face-like stimuli shortly after birth. This model provides an excellent example of the developmental concept of constraints by design. Newborn visual preferences are proposed to be largely determined based on perceptual constraints associated with the immature visual system as well as binocular correlations that occur based on the structured relations between the spacing and location of the newborn’s eyes and general characteristics of faces and face-like patterns. Future research in the area should continue to utilize computational modeling simulations to test the feasibility of additional factors that may influence newborn attentional biases. We now turn our focus to the development of face processing across the infancy period.

## Development of Face Processing in Infancy

A large body of work has been carried out on the development of face processing in infancy. We propose that findings from the extant literature demonstrate the important role of *experiential canalization* (Kuo, 1976; Gottlieb, 1991; Lickliter and Harshaw, 2010) in the development of face processing. Experiential canalization refers to the concept that development is a cumulative process. As development proceeds, the range of behavioral potentials or plasticity narrows. This decrease in plasticity is driven by experience and the developmental history of the organism. Several lines of research provide examples of experiential canalization by demonstrating the effects of regular postnatal exposure to certain types of faces on the development of face processing across the infancy period.

Research indicates that minimal exposure is required for newborn infants to develop a preference for their mother’s face (Pascalis et al., 1995; Sai, 2005). For example, Field et al. (1984) found that 45-hour-old infants showed a preference for their mother’s face over a stranger’s face with an average of just four discontinuous hours of interaction with their mothers for feedings before testing. Somewhat surprisingly, the authors found that newborns habituated to their mother’s face with repeated exposure and showed novelty preferences for a stranger’s face on later testing trials. Similarly, Barrera and Maurer (1981) found that 3-month-olds show an initial preference for the mother’s face over a stranger’s face followed by a shift to a novelty preference for the stranger’s face on subsequent testing trials. These findings indicate that experience is a driving force behind these early face preferences. Although only an average of 4 h of exposure to their mother’s face was needed for infants to demonstrate visual preferences for their mother’s face (Field et al., 1984), further exposure within the testing context led to a shift to looking longer to the stranger’s face. Farroni et al. (2013) utilized functional near infrared spectroscopy (fNIRS) to measure the cortical hemodynamic response of 1- to 5-day-old newborns in response to dynamic faces, arms, and moving objects. The results indicated face specific activation of bilateral posterior temporal cortex that was positively correlated with age in hours. Thus, at both the behavioral and neural levels, even very young infants’ responses to faces are highly malleable and can change with very limited experience.

A large body of research has shown that infants show a preference for female faces in early development that is most likely due to heavy exposure to their mother or a female caregiver. A study of 3- to 4-month-old infants familiarized with both female and male faces found that infants consistently displayed a preference for female faces (Quinn et al., 2002). However, infants primarily raised by males demonstrate preferences for male faces over female faces (Quinn et al., 2002), indicating that gender preference is based on extensive experience with the infant’s primary caregiver. In a subsequent study, Quinn et al. (2008) tested 3-month-old Caucasian infants and found they preferred female Caucasian faces over male faces, but did not prefer female over male faces when the faces were Asian. Thus, infant preference for female faces is specific to the race of the mother and is not present when the female face is of another race. Additionally, when testing newborn Caucasian infants, the participants did *not* show a preference for female Caucasian faces, further supporting the theory that it is repeated experience with their mother that biases infants’ facial preference.

There has been a significant amount of research conducted on development of the other-race effect (ORE) in infancy. This effect refers to a disadvantage for processing and recognizing individual exemplars of other-race faces in comparison to own-race faces (Hugenberg et al., 2010). Sangrigoli and de Schonen (2004) found that 3-month-olds demonstrate evidence of the ORE that can be eliminated with very brief exposure to other-race faces. Kelly et al. (2007, 2009) found that the ORE increases from 3 to 9 months of age. However, the ORE is not found for infants: raised in environments in which they experience regular exposure to other-race faces (Bar-Haim et al., 2006), regularly shown picture books with other-race faces (Heron-Delaney et al., 2011), or given brief daily exposure to dynamic other-race faces (Anzures et al., 2012). Research utilizing eye-tracking indicates that across 4–9 months of age, infants develop differential scanning patterns for own- vs. other-race faces that coincides with decreased recognition memory ability for other-race faces (Liu et al., 2011; Wheeler et al., 2011; Xiao et al., 2013).

The effects of repeated exposure to certain types of faces has been studied more broadly in research on perceptual narrowing examining infant perception of “native” vs. “non-native” faces. Perceptual narrowing is a developmental process that occurs as infants gain extensive experience with stimuli specific to their native environment (Pascalis et al., 2002; Scott et al., 2007; Lewkowicz and Ghazanfar, 2009; Maurer and Werker, 2014). As the narrowing process unfolds infants transition from having perceptual sensitivities that are broadly tuned to a wide range of stimuli to being more narrowly focused on the stimuli encountered regularly in the native environment. Thus, perceptual narrowing can be viewed as a form of experiential canalization. The other-species effect (OSE) is an example of perceptual narrowing in face processing. The body of behavioral research on this effect suggests across the 6–9 months age range, there is maintenance of perceptual sensitivity for own-species faces and a decrease in perceptual sensitivity for other-species faces (e.g., Pascalis et al., 2002; Simpson et al., 2011). Simpson et al. (2011) proposed a learned attention model of face processing that states that with age and increased experience, infants learn to focus their attention on facial dimensions useful for identification of own-species faces encountered in their everyday experience. The proposal that learned attention drives perceptual narrowing in face processing is consistent with research showing that infants trained with picture books of individually labeled monkey faces between 6 and 9 months of age maintain the ability to individuate other-species faces at 9 months of age (Pascalis et al., 2005; Scott and Monesson, 2009, 2010). In contrast, control infants who receive no supplemental exposure to monkey faces, or infants that receive training with picture books without individually labeled monkey faces do not demonstrate the ability to individuate monkey faces at 9 months of age. Thus, the verbal pairing of individual labels with faces seems to have a significant influence on the maintenance of infants’ sensitivity to own-species faces, again indicating the multimodal stimulation is an important aspect of early cognitive development and perceptual learning.

## Neural Correlates of Infant Face Processing

In addition to behavioral research, there has been extensive developmental cognitive neuroscience research done on face processing and perceptual narrowing in infancy. The event-related potential (ERP) has been widely used in research on face processing in both infants and adults. The N290 and P400 are two ERP components that have been shown to be associated with face processing in infancy (de Haan et al., 2003; de Haan, 2007). The N290 is commonly identified at posterior electrodes between 290 and 350 ms after stimulus onset (Halit et al., 2003), and is greater in amplitude to faces than noise by 3 months of age (Halit et al., 2004). The P400 is also commonly found at posterior electrodes between 390 and 450 ms after stimulus onset. The P400 has a shorter latency to peak in response to faces than objects by 6 months of age (de Haan and Nelson, 1999), and a shorter latency to upright vs. inverted human faces (Halit et al., 2003) by 12 months of age. Stimulus inversion is used in face processing research to examine the development of configural processing in faces. Configural processing represents more advanced processing of faces in comparison to featural processing (Maurer et al., 2002). An impairment in processing inverted faces compared to upright faces is used as a marker for configural processing of face stimuli (Yovel and Kanwisher, 2005; Rossion and Curran, 2010). Thus, these findings indicate a trend across infancy toward configural processing of own-species faces.

Although 9-month-old infants do not typically show inversion effects in ERP responding to monkey faces (Scott et al., 2006), Scott and Monesson (2010) found that 9-month-olds given 3 months of training with pictures of monkey faces labeled at the individual level demonstrate both N290 and P400 inversion effects for inverted compared to upright monkey faces. This finding, coupled with the finding that 9-month-old infants given similar training can demonstrate behavioral evidence of discriminating monkey faces at the individual level shows the positive effects of pairing faces with verbal labels on the maintenance of infants’ sensitivity to other-species faces (Pascalis et al., 2005; Scott and Monesson, 2009). Thus, augmented experience through extensive training has a positive impact on infants’ ability to maintain sensitivity to other-species faces.

Research from our lab (Dixon et al., 2017), has shown that although infants demonstrate poor performance at individuating monkey faces based on perceptual narrowing, they do seem to be efficient at categorizing other-species faces at 9 months of age. In addition to analyzing face processing components, we analyzed the Negative central (Nc) ERP component associated with infant attentional engagement (Courchesne et al., 1981; de Haan and Nelson, 1997, 1999, Reynolds and Richards, 2005; Reynolds et al., 2010; Reynolds, 2015; Reynolds and Romano, 2016). We found that with a training phase that consisted of only 20 brief presentations of multiple exemplars of monkey faces from a specific monkey species (e.g., Capuchin monkeys), 9-month-olds were able to demonstrate strong evidence of subordinate-level categorization of other-species faces. Subordinate-level categorization is considered to be a marker for perceptual expertise (Quinn and Tanaka, 2007).

**FIGURE 1:**
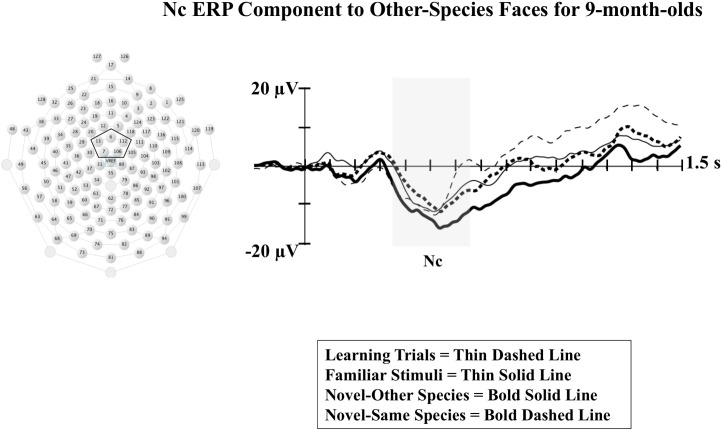
The Nc component associated with infant visual attention is shown at midline central electrode clusters for 9-month-old infants in response to presentations of monkey faces that were either: familiar faces shown during an initial learning phase (first 20 trials), novel monkey faces from a different species than those seen in the learning phase (novel-other), or novel faces from the same species as those seen during the learning phase (novel-same). The midline central electrode cluster used in the analyses is indicated in the sensor net layout shown to the left. The shaded rectangle indicates the time window for the analysis of Nc. Time following stimulus onset is shown on the *X*-axis, and change in amplitude of the ERP (in microvolts) is shown on the *Y*-axis (Figure adapted from Dixon et al., 2017).

Results from our analysis of Nc are shown in **Figure 1**. Infants demonstrated greater Nc amplitude to monkey faces from a different species of those they were trained on (novel-species condition) compared to Nc amplitude for both novel monkey faces from the same species they were exposed to during training (novel-same) and the familiar faces seen during training (familiar). The amplitude of Nc during the training trials is also shown. This finding is indicative of increased attention to the novel-species monkey faces, and provides evidence of subordinate-level categorization for other-species. Although further studies are needed examining categorization of other-species faces across a broader age range, these findings provide some support for the possibility that selective attention may serve as a mechanism behind perceptual narrowing. By 9 months of age, infants may be distributing their selective attention during initial exposure to non-native stimuli in a manner effective for processing at the categorical level (i.e., “what is this?”) as opposed to the individual level (i.e., “who is this?”). A visual intake strategy aimed at categorization as opposed to individuation would be the most efficient initial approach to perceptual processing of a novel species not encountered in the native environment.

The bulk of the extant literature thus indicates that experience plays a pivotal role in the process of developing attentional biases in early infancy. These biases subsequently affect the development of face processing expertise and social preferences in later infancy. Frequent exposure to the same types of stimuli, such as faces of the same gender and race as their primary caregiver in early development (Quinn et al., 2002, 2008) lead to basic familiarity preferences. Over time and with experience, these preferences translate to attentional biases that serve to facilitate processing of native stimuli at finer levels of discrimination (i.e., individuation) than non-native species (categorization). These findings demonstrate experiential canalization in the development of face processing and indicate that the effects of prior experience on developing face processing abilities may be mediated by differential distribution of selective attention for familiar compared to novel stimulus classes.

## The Development of Face Processing in Multimodal Contexts

Across studies reviewed above, infants were tested in unimodal visual conditions. However, social experience with faces is multimodal and often involves combined sensory input to the visual and auditory sensory modalities in the form of audiovisual speech. In the next section, we review research examining infant processing of faces in multimodal contexts that indicates characteristics of multimodal sensory stimulation play a critical role in directing infant attention and facilitating perceptual learning. We propose that in addition to highlighting the role of experiential canalization in early face processing, findings from this research provide an excellent example of the developmental concept of *distributed control* (Lickliter, 2000; Lickliter and Honeycutt, 2003). Distributed control indicates that the control of developmental outcomes is distributed across levels of the developmental system, and is determined by relations within and between organismic and contextual variables (Lickliter, 2000). Under this framework, no single factor is viewed as having causal priority in determining developmental outcomes. Instead, developmental outcomes are reciprocally determined based on the interdependent and mutually constraining influence of multiple factors (Oyama, 1985, 1993; Gottlieb, 1991, 1997; Lickliter, 2000; Lickliter and Honeycutt, 2003).

There is a large body of research demonstrating that multimodal stimulation is a highly salient source of information that serves to engage attention and facilitate perceptual processing and memory in human infants, human adults, and precocial avian species (Bahrick and Lickliter, 2000, 2002, 2012, 2014; Lewkowicz, 2000; Lickliter and Bahrick, 2000; Reynolds and Lickliter, 2004; Reynolds et al., 2013, 2014). Bahrick and Lickliter’s (2000, 2002, 2014) intersensory redundancy hypothesis proposes that redundancy across two or more sensory systems directs selective attention to amodal properties of objects and social events that are perceived across multiple sense modalities. This focus of selective attention on the amodal properties of multimodal stimuli occurs at the expense of non-redundantly specified, modality-specific stimulus properties. Thus, in the context of face processing, intersensory redundancy would be expected to facilitate processing of amodal information provided by faces (e.g., affect, prosody; Flom and Bahrick, 2007), and interfere with processing modality-specific information provided by faces (e.g., distinctive facial features used for individuation and face recognition; Bahrick et al., 2013). The role of intersensory redundancy in directing selective attention and promoting perceptual learning of amodal information is proposed to be most important in early development when attentional resources and prior experience are both highly limited.

Flom and Bahrick (2007) conducted a series of habituation experiments examining the ability of infants to discriminate a change of affect conveyed through speech. Infants of 3, 4, 5, or 7 months of age were shown video clips a woman speaking with either a happy, sad, or angry affective expression. These video clips were presented in the following conditions: synchronous (redundant) audiovisual, asynchronous (non-redundant) audiovisual, unimodal auditory, or unimodal visual. Results showed that at 4 months of age, infants were only able to discriminate a change in affect in the synchronous audiovisual condition. By 5 months of age infants were able to detect the change in the unimodal auditory condition. Infants were only able to detect the change in affect in the unimodal visual condition at 7 months of age. Asynchronous audiovisual presentation was found to interfere with infants’ detection of a change in affect. These findings provide an example of intersensory facilitation in that infants detected changes in affect in redundant audiovisual speech at an earlier age than in unimodal speech. Similarly, Coulon et al. (2013) found evidence of intersensory facilitation of neonatal imitation of mouth movements conveyed in audiovisual speech. Newborns imitated mouth movements produced by a model in an audiovisual congruent condition more quickly than in a unimodal visual condition. Furthermore, newborns failed to imitate mouth movements produced by a model in an audiovisual incongruent condition.

Face recognition relies on successful processing of facial features which are specific to the visual modality. If intersensory redundancy directs infant attention to amodal stimulus properties at the expense of modality-specific stimulus properties, then infants should show facilitation of face recognition under unimodal stimulus presentations in comparison to redundant multimodal stimulus presentations. In support of this prediction from the intersensory redundancy hypothesis, Bahrick et al. (2013) found infants were able to discriminate novel from familiar faces in a unimodal visual condition at 2 months of age; however, infants did not show evidence of discriminating novel from familiar faces in a synchronous audiovisual condition until 3 months of age. Consistent with the concept of distributed control, these findings indicate infant performance on measures of face processing is dependent on multiple factors; including (but not limited to): age, stimulus modality, and whether successful performance on the task relies on detection of amodal or modality-specific stimulus properties. Furthermore, the results of these studies imply that research on infant face processing that utilizes static visual stimuli may not generalize well to infant face processing of dynamic faces in multimodal contexts.

Bahrick et al. (2016) examined 2- to 8-month-old infants’ attention to faces compared to objects under static and dynamic audiovisual and unimodal visual presentation conditions. Interestingly, they found no attentional bias for faces compared to objects for infants at 2 months of age. By 3 months of age, infants only attended more to faces compared to objects under dynamic presentation conditions, and from 4 months on, infants began to focus more on dynamic audiovisual faces compared to all other stimulus types. The authors concluded that enhanced selective attention to faces compared to non-social stimuli emerges gradually across infancy. Bahrick et al. (2013) have also proposed that infants are not innately attracted to faces over other sources of information in early infancy. In contrast, motion or action and intersensory redundancy are proposed to be higher on the salience hierarchy in early infancy in comparison to faces *per se*. Support for this hypothesis comes from research indicating that infants demonstrate poor face perception when faces are seen in naturalistic settings. For example, 5-month-olds shown videos of an actress engaged in a repetitive action (such as brushing her hair) show discrimination and evidence of long-term memory for the action, but they show no evidence of discrimination or memory for the actress unless the length of exposure is doubled or the repetitive action is eliminated (Bahrick et al., 2002; Bahrick and Newell, 2008). Thus, in dynamic, multimodal contexts, infants may be biased to focus selective attention on motion (or action) first, and then shift selective attention to stimulus properties lower on the salience hierarchy after each of the more salient properties is processed. Frank et al. (2009) utilized eye-tracking to examine the distribution of 3- to 9-month-old infants’ selective attention while viewing animated films. They found that 3-month-olds’ selective attention was driven by low-level stimulus salience, and infants gradually began to focus more on faces beyond 3 months of age. Taken together, these findings indicate that faces move to higher levels in the salience hierarchy compared to non-social stimuli from 4 months on (Frank et al., 2009; Bahrick et al., 2013).

Lewkowicz and Ghazanfar (2006), Lewkowicz and Hansen-Tift (2012), Lewkowicz (2014), and Minar and Lewkowicz (2017) have examined perceptual narrowing in audiovisual speech perception. For example, Lewkowicz and Hansen-Tift (2012) utilized infrared eye-tracking to investigate infant looking patterns across an age range of active language learning. Video clips of women speaking either English (native language) or Spanish (non-native language) were shown to 4 - to 12-month-old English-learning infants as well as monolingual English-speaking adults. Both adults and 4-month-olds looked mostly at the eyes of a talking face, but starting at 6 months of age, infants began to look more at the mouth of the speaker regardless of what language was being spoken. Around 10–12 months of age, infants listening to the English speaker began to shift the overall distribution of their selective attention such that relatively more looking was focused toward the mouth again, much like adults. But 10 and 12-month-olds listening to a woman speak in Spanish remained focused on the mouth to a greater extent.

These results indicate that in the youngest group at an earlier stage of language development, infants selectively attended to the mouth as a source of redundant information provided by the vocalizations and movements of the mouth. By 10–12 months of age, infants learning English have a more mature language foundation and may not need to rely on the mouth movements as heavily to process the audiovisual speech. However, the older infants listening to the non-native Spanish speaker may still require the redundant information presented by the mouth of the speaker. Interestingly, Kubicek et al. (2014) found that although 6-month-olds are capable of cross-modal matching of audio and video tracks of a woman speaking in both native and non-native speech, 12-month-olds are only able to do so with non-native speech. Although this finding seems counter-intuitive in the context of perceptual narrowing, the 12-month-olds’ poor ability to match face and voice in the native speech condition may have been based on increased selective attention to the eyes relative to the mouth for native language speakers (Lewkowicz and Hansen-Tift, 2012). Thus, in the native speech condition, 12-month-olds may have not focused their selective attention on the redundant properties of speech provided by the mouth and this may have decreased their ability to engage in cross-modal matching.

In a recent study, Minar and Lewkowicz (2017) found that infants rely on multimodal cues for discriminating other-race faces. By 10–12 months of age, infants were only able to discriminate other-race faces when presented in a synchronous audiovisual condition. Furthermore, while they were able to discriminate own-race faces in a unimodal visual condition, they were unable to discriminate other-race faces in the unimodal visual condition. Taken together, these findings are in line with the most recent tenet of the intersensory redundancy hypothesis which proposes that older infants and children revert to relying on intersensory redundancy to facilitate perceptual processing and learning in more challenging contexts (Bahrick et al., 2010), and they demonstrate the effects of experience on intersensory perceptual narrowing.

## Neural Correlates of Infant Multimodal Perceptual Processing

In addition to behavioral measures, studies have also utilized neural measures to examine infant audiovisual processing. Hyde et al. (2010) conducted an ERP study examining unimodal and multimodal speech processing in 3-month-olds and adults. Participants were presented an actress saying “hi!” in infant-directed speech in unimodal auditory, unimodal visual, and bimodal audiovisual conditions. Results indicated that 3-month-olds demonstrate an enhanced N450 ERP component over fronto-temporal sites during bimodal audiovisual presentations. The N450 ERP component is considered a precursor to the N2 component, which is associated with auditory processing in adulthood (Wunderlich and Cone-Wesson, 2006). This suggests that simultaneous visual stimulation facilitates auditory processing in early infancy continuing into adulthood and is consistent with findings of super additive multimodal effects on neural activity from comparative research (Jay and Sparks, 1984; Stein et al., 1994; Wallace et al., 1996; Wallace and Stein, 1997) and research with adults (e.g., Giard and Peronnet, 1999; Santangelo et al., 2008).

Several studies have examined the effects of audiovisual face-voice pairings on the Nc component associated with infant visual attention (Grossmann et al., 2006; Hyde et al., 2011; Vogel et al., 2012; Reynolds et al., 2014). In two initial studies, infants’ audiovisual integration was tested by examining neural responsiveness to test stimuli presented that were either congruent or incongruent in affect with a preceding stimulus. Using this approach, Grossmann et al., (2006) found that infants demonstrate greater Nc amplitude to face-voice pairings conveying incongruent emotional information compared to face-voice pairings conveying congruent emotional information. Yet, Vogel et al., (2012) found that infants demonstrate greater amplitude Nc to face-voice pairings conveying congruent emotional information. These contrasting findings may have been due to procedural differences that could potentially alter the salience hierarchy of congruent vs. incongruent stimuli across studies. This would lead to differences in the directional effects of Nc as it is associated with depth of attentional engagement (Reynolds et al., 2010). Because the auditory and visual components of the stimuli used in these studies were not presented simultaneously, these studies did not provide insight into the neural underpinnings of the effects of intersensory redundancy on attention and perceptual processing in infancy.

Two studies have examined the effects of intersensory redundancy on neural correlates of infant attention and memory in response to audiovisual speech (Hyde et al., 2011; Reynolds et al., 2014). Both of these studies tested infants at 5 months of age, and analyzed Nc as an index of attentional engagement. The late slow wave (LSW) was examined as a neural correlate of infant recognition memory. Across studies, infants have been found to demonstrate reduced amplitude of the LSW with increased stimulus exposure (de Haan and Nelson, 1999; Snyder et al., 2002, 2010; Wiebe et al., 2006; Guy et al., 2013). Hyde et al. (2011) found greater amplitude of the LSW on synchronous audiovisual trials in comparison to asynchronous audiovisual trials, and interpreted this finding to indicate enhanced processing of synchronous audiovisual speech. In contrast, infants demonstrated greater amplitude Nc on asynchronous audiovisual trials in comparison to synchronous audiovisual trials. The authors interpreted this as increased infant attention to the novelty of speech presented asynchronously across the auditory and visual modalities.

**FIGURE 2:**
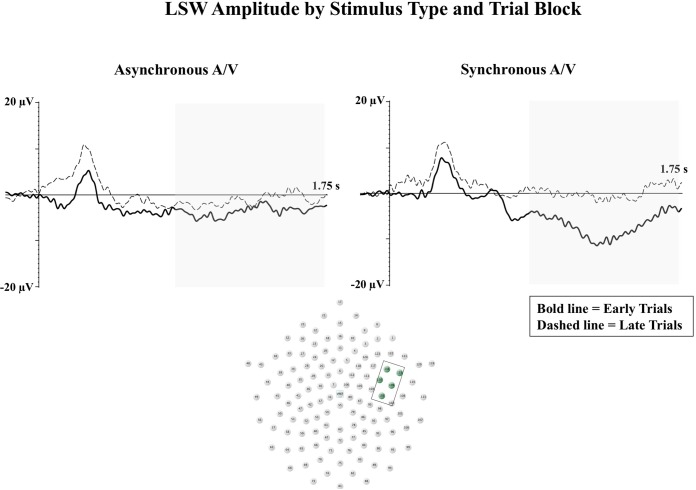
The LSW is shown for early (bold line) and late (dashed line) blocks of trials at right anterior temporal electrodes. Waveforms from the synchronous audiovisual condition are shown in the right panel, and waveforms from the asynchronous audiovisual condition are shown in the left panel. The *Y*-axis represents the amplitude of the ERP in microvolts, and the *X*-axis represents time following stimulus onset. The time-window of the component analysis is shaded on the *X*-axis. The positioning of the electrodes included in the analysis are shown in the bottom panel (Figure adapted from Reynolds et al., 2014).

Reynolds et al., (2014) conducted two ERP experiments examining 5-month-old infant visual attention and recognition memory for speech presented in unimodal visual, synchronous audiovisual, and asynchronous audiovisual conditions. The first experiment examined the effects of intersensory redundancy on attentional engagement. In contrast to Hyde et al. (2011) findings, infants were found to demonstrate greater amplitude Nc to synchronous audiovisual speech in comparison to asynchronous audiovisual or unimodal visual speech. Once again, these contrasting findings in the direction of Nc effects could be due to procedural differences. As the Nc reflects level of attentional engagement, variations in testing context would be expected to affect infant attention and relative amplitude of the Nc component (Richards, 2003). Importantly, both studies demonstrated LSW activity associated with enhanced perceptual processing on synchronous audiovisual trials. For example, Reynolds et al. (2014) utilized a block design in their second experiment to examine changes in LSW amplitude from early to late trials. Infants only demonstrated significant reductions in LSW amplitude from early to late trials in the synchronous audiovisual condition (see **Figure 2**). These findings indicate that the intersensory redundancy provided in the synchronous audiovisual condition led to enhanced infant attention (greater amplitude Nc) and enhanced perceptual processing resulting in recognition memory in the late block of trials (reduced amplitude LSW).

Taken together, the findings from a growing body of research utilizing both behavioral and neural measures indicate that infant selective attention and perceptual processing of amodal information is enhanced in multimodal testing conditions in comparison to unimodal testing conditions. However, consistent with the development of distributed control, the interaction of multiple factors (e.g., age, stimulus modality, previous experience) determines which stimulus properties an infant will focus selective attention on and ultimately process. After birth, infants are immersed in social environments. Sugden et al. (2014) utilized head mounted cameras on 1- and 3-month-old infants in their home environments, and found that 25% of the infants’ waking time was spent exposed to faces. Research indicates that the distribution of selective attention to faces shows significant developmental change across infancy. This developmental change is likely tied to a number of factors, including (but not limited to) extensive experience with faces, individual differences, and early language development (e.g., Kushnerenko et al., 2013; Tomalski et al., 2013; Streri et al., 2016). In addition to the effects of prior experience on shaping attentional biases in early development, there are likely a number of neural and physiological mechanisms that influence attention to faces during infancy.

## Neural and Physiological Mechanisms Involved in Attentional Biases

Attention and arousal are tightly linked in early development. Comparative research (e.g., Radell and Gottlieb, 1992; Reynolds and Lickliter, 2004) and research with human infants (e.g., Gardner and Karmel, 1995; Geva et al., 1999) has shown that alterations in physiological arousal can modulate attention and either enhance or interfere with perceptual learning and responsiveness in early development. For example, Gardner and Karmel (1995) found that both internally and externally induced arousal modulates infant selective attention such that newborn infants focus their attention on low frequency sine wave stimuli during high arousal states and they focus their attention on high frequency stimuli during low arousal states. Similarly, newborns’ visual preferences in the paired-comparison task were found to shift from familiarity preferences when tested prior to feeding to novelty preferences after feeding (Geva et al., 1999). Blass and Camp (2001) tested 9- and 12-week-old infants for evidence of recognition of a research assistant. Initial exposure to the research assistant was either paired with delivery of a sucrose solution or not. The authors found that in subsequent testing, infants only demonstrated recognition of the research assistant when initial exposure was paired with the sweet taste of the sucrose solution. Additionally, only infants who were calm during testing demonstrated evidence of recognition memory.

Arousal-based effects on infant visual attention are strongest in the newborn period, and Gardner and Karmel (1995) have proposed they serve to maintain an optimal level of arousal for the child in the earliest stages of postnatal development. In comparative work with Bobwhite Quail embryos and chicks, Reynolds and Lickliter (2004) found that significant alterations in arousal associated with prenatal sensory stimulation have prolonged effects on arousal and perceptual processing that persist into early postnatal development. Several studies have demonstrated the importance of the primary caregiver for the regulation of arousal in young infants (e.g., Gable and Isabella, 1992; Calkins, 1994; Porter, 2003), indicating infants are dependent on caregivers for other-regulation during the early months of postnatal development. Gredebäck et al. (2012) utilized eye-tracking to examine fixation patterns and pupil dilation in a study on individual differences in face processing in infancy. They found that parental care influenced both gaze duration and pupil dilation for 14-month-olds viewing images of their parents or strangers. Specifically, infants who received similar levels of parental care from both their mother and father showed more broadly distributed gaze patterns than infants primarily cared for by their mother. Furthermore, infants showed larger pupil diameter when viewing images of their secondary caregiver displaying neutral affect. Thus, a promising direction for future research would be to examine arousal-based mechanisms that may be associated with the development of social orienting and face processing.

Arousal – attention relations are bidirectional. For example, attentional engagement leads to changes in arousal in infancy (for review, see Reynolds and Richards, 2008). Richards (2008), Reynolds et al. (2013), and Reynolds and Romano (2016) have proposed that there is a general arousal/attention system that accounts for the effects of attention on various aspects of arousal. Several areas of the brain contribute to this general arousal/attention system including, brainstem areas, thalamus, basal forebrain, and cardio-inhibitory centers in frontal cortex (Reynolds et al., 2013). The cholinergic system is also critically involved in sustained attention (Sarter et al., 2001). Activation of this system has a range of effects related to arousal, including: decreased heart rate, decreased motor activity, and release of acetylcholine (ACh) via corticopetal projections. These changes foster an optimal level of arousal for attention and perceptual processing. This arousal system shows considerable development across infancy and early childhood. Developmental changes associated with further development of the general arousal/attention system include: increased magnitude of the heart rate response associated with attention, longer durations of sustained attention, and decreases in distractibility across infancy and early childhood (Richards and Cronise, 2000; Reynolds and Richards, 2008). Guy et al. (2016) utilized heart rate, ERP, and cortical source analysis to examine face processing in 4.5- to 7.5-month-old infants. Their findings indicated that ERP components associated with infant face processing (i.e., N290, P400) were greater in amplitude on trials when heart rate was indicative changes in arousal associated with attention. Additionally, the results of the source analysis revealed occipital–temporal areas, such as the middle fusiform gyrus, as a potential source of the N290 ERP component.

Multiple brain networks show further development throughout infancy that have a significant influence on the characteristics of infant attention to both social and non-social stimuli. For example, at birth attention is primarily influenced by subcortical structures, including the superior colliculus. During the newborn period, visual fixations are believed to be primarily reflexive (Johnson et al., 1991c), and infant visual attention is reflexively drawn to areas of high-contrast in the visual field, motion, and stimuli that are larger in size. This subcortical reflexive system is consistent in many ways with Conspec (Johnson and Morton, 1991b); however, this system is domain-general as opposed to an innate system evolved for orienting to faces. It is not until about 2–3 months of age that areas of the brain involved in the voluntary control of visual fixation begin to reach functional onset, these include posterior parietal areas, the pulvinar nucleus of the thalamus, and frontal eye-fields (Posner and Petersen, 1990; Johnson et al., 1991c; Petersen and Posner, 2012). Finally, beyond 6 months of age, frontal areas (dorsolateral prefrontal cortex, orbitofrontal cortex, anterior cingulate) have a greater influence on attention. Further development of these frontal areas as well as increased frontal – parietal and frontal – temporal connectivity contribute to gains in the volitional control of attention, and increased inhibition to distracters. These developmental changes are likely tied to gains in social – cognitive processes, such as categorization of social agents and comprehension of the actions of social agents (Grossmann, 2015).

## Limitations and Future Directions

Although a great deal of progress has been made in research on the development of face processing, controversy still remains regarding the mechanisms that account for both newborn face preferences and for the rapid development of relatively advanced face processing ability across the infancy period. The vast majority of research in the area has utilized cross-sectional designs. In order to gain insight into processes involved in the development of attentional biases for faces, more longitudinal studies need to be carried out across relatively broad age ranges. Additionally, scientists have been somewhat limited in neuroimaging tools that are available for use in research on early development given practical and ethical concerns related to the use of fMRI and PET with infant participants. To gain greater understanding of both neural processes and neural systems involved in early face processing, future studies should be aimed at capitalizing on: the excellent temporal resolution of ERP, the advanced spatial resolution of fNIRS, and the added level of insight provided by computational modeling.

## Conclusion

We propose that developmental systems theory provides an ideal framework for interpreting the development of attentional biases for faces in infancy. The extant findings from research on infant processing highlight the cumulative nature of development and are consistent with the concept of experiential canalization (Kuo, 1976) in that early experience serves to direct subsequent experience. It is our position that biases to attend to faces are not innately determined or set at birth, but instead are the product of domain-general developmental processes. The distribution of selective attention is determined by multiple influences; including previous experience, stimulus characteristics, arousal mechanisms, and the functional maturity of brain structures involved in attention. Consistent with the developmental concepts of constraints by design and distributed control (Lickliter, 2000), it is the interaction of these multiple factors that determines how selective attention is distributed at any given point in development. None of these factors are viewed as having causal priority in determining infant visual preferences; however, the relative influence of each factor will change across contexts and further development. For example, the influence of arousal on attention decreases beyond the newborn period (Gardner and Karmel, 1995), and the influence of experience and learning increases throughout early development as is demonstrated through the effects of perceptual narrowing. The process of learning through experience, combined with further development of neural systems involved in attention and cognitive processing, allows the infant greater efficiency and flexibility in social – cognitive processing from late infancy on.

## Author Contributions

GR determined a general outline for the review paper and came up with the model presented in the paper regarding the development of attentional biases in infancy. After the authors discussed the general outline of the paper, KR wrote two initial drafts with feedback from GR. GR then wrote the submitted version of the paper utilizing portions of the initial drafts written by KR, as well as adding additional sections and adding the final model presented in the paper.

## Conflict of Interest Statement

The authors declare that the research was conducted in the absence of any commercial or financial relationships that could be construed as a potential conflict of interest.
